# Validating the preeminence of biochemical properties of camel over cow and goat milk during the Covid‐19

**DOI:** 10.1002/fsn3.2881

**Published:** 2022-04-29

**Authors:** Amal M. AL‐Saffar

**Affiliations:** ^1^ Department of Biological Sciences Faculty of Science, Kuwait University Kuwait Safat Kuwait

**Keywords:** biochemical properties, camel, cow, goat milk, Kuwait, trace metals

## Abstract

In the light of the Covid‐19 pandemic outbreak, and the need‐of‐the‐hour to boost immunity to residents, especially those residing in an arid environment, a comparative study was made on the physical and biochemical properties of dairy milk. This novel study in Kuwait revealed the lesser consumed pseudoruminant camel milk as a better potential source of dietary inclusion and an immune booster over true ruminants—cow's and goat's milk. Analysis using a wide array of instruments determining the physical characteristics in camel's milk (pH, conductivity, specific gravity, moisture, and total solids), biochemical constituents (crude protein (CP), nonprotein (NP), and fat), and inorganic constituents (K‐919; Ca‐907; Zn‐4.2 mg/100 mg) revealed conducive properties that validate immunity to consumers when compared to the regularly used cow's milk (K‐841; Ca‐776; Zn‐2.43 mg/100 mg) and goat's milk (K‐914; Ca‐849; Zn‐2.45 mg/100 mg). Log‐transformed results revealed high vitamin C in camel's milk (0.42 mg/100 g), indicating high antioxidant properties compared to those of goat's milk (0.12 mg/100 g) and cow's milk (0.04 mg/100 g). Statistical tests by analysis of variance (ANOVA) revealed significant differences and the correlation coefficient between the three milk samples validating the multiple reasons to use camel's milk over the cow's and goat's milk. Furthermore, this study recommends the consumption of camel's milk due to its low concentrations of contaminants as well, their status below permissible limits in Kuwait, set by global standards over the other sampled milks.

## Graphical abstract

Following the analysis of three milk samples, this study revealed a novel validation to camel's milk edging over cow's and goat's milk in their elemental and biochemical properties, thus recommending the camel milk as an immune booster in the wake of the Covid‐19 pandemic.

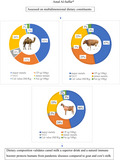

## 1 INTRODUCTION

Milk, an exceptional source of vital nutrition for the human diet, is mostly composed of the bioavailable calcium, phosphorus, fat, protein, several essential metals, and vitamins in a balanced ratio than the other foods (Hossain & Dev, 2013). Milk from various mammals is converted by man to different nutritionally enriched dairy products. The camel is one such mammal that enhances the socioeconomic significance through its milk and is an important part of human diets in many arid and semiarid regions of the world. Evidence revealed the characteristic presence of antimicrobial properties in camel milk that is medicinally used to combat human diseases (Kaskous, 2016; Singh et al., 2017; Zibaee et al., 2015). However, evidence seldom probed their importance and use during the Covid‐19 pandemic outbreak, although the camel milk experiences have created a novel awareness on their use in the Western world, ever since the Food and Agricultural Organization (FAO) promoted the camel's milk business (Mirzaei, 2012).

Globally, the human's favorite choice of the palate is the milk of cow and goats, and hence, the nutritional and economic well‐being of humans is tremendous from the contribution of cow and goat milk. These milks have several characteristics that are attributed to innumerable nutritional and health benefits (Alcantara et al., ; El‐din, 2012; Legesse et al., 2017; Turkmen, 2017; Zenebe et al., 2014). Comparatively, goat's milk has a higher nutritional value than cow's milk. However, the cow's milk is widely used because of people being habituated to drinking milk, voluminous production, lower market price, and organized global apportionment of milk and its dairy products (Statista, 2019). Globally, the consumption of camel milk is limited, although studies reveal excellent antioxidant and antimicrobial properties, lactoferrin content resulting in low citrate concentrations, and high level of immunoglobulin G (IgG) (1.64 mg/L) compared to the IgG in cow's (0.67 mg/L) and goat's (0.7 mg/L) milk (Tahereh & Hussain, 2021). Nevertheless, the merits of these milks were shown, the adverse effects on human health and a serious threat to food safety were found to have developed into a worldwide significant issue because of inorganic pollutants such as heavy metals in soil and plants (Muhib et al., 2016). The trophic transfer of metals followed the food chain from plants, soil, water, and anthropogenic sources through milking animals to humans (Ali & Khan, 2019; Boudebbouz et al., 2021; Chirinos‐Peinado & Castro‐Bedriñana, 2020).

Essential metals (potassium (K), calcium (Ca), sodium (Na), magnesium (Mg), iron (Fe), manganese (Mn), copper (Cu), zinc (Zn), sulfur (S), selenium (Se), and cadmium (Cd)) were <20 µg/L and nonessential metals (aluminum (Al), vanadium (V), molybdenum (Mo), mercury (Hg), arsenic (As), silver (Ag), and lead (Pb)) were <10 µg/L in milk. These metals became toxic at high concentrations (>10 µg/L; Amir et al., 2017). These metals were found to be toxic and nondegradable even at a low concentration (1.0–10.0 mg/L; Ayangbenro & Babalola, 2017). Significant quantities of metals are found to have been transferred from contaminated soil to plants, causing the accumulation of potentially toxic metals in grazing ruminants. Accumulation of metals in ruminants causes toxic effects not only in cattle, but also in humans consuming meat and milk contaminated with toxic metals (Mason et al., 2014; Mohsin et al., 2019; Pilarczyk et al., 2013). Earlier studies (by Ping et al., 2014; Sarsembayeva et al., 2020; Shahbazi et al., 2016) fortified the permissible levels of heavy metals in milk through the joint Food and Agriculture Organization (FAO)/World Health Organization (WHO) *Codex* *Alimentarius* Commission and observed the harmful effect of high trace metals’ concentrations through milk consumption by humans. Among the prevalent three types of milk, the quality of camel's milk from the local supply was suspected of metals bioaccumulation and exceeding the permissible limits, although immunity properties such as the high levels of vitamin C, high immunoglobulins (IgG), and low citrate levels were observed (Faye et al., 2019; Tahereh & Hussain, 2021). Thus, the present study corroborates the impact from the recent Covid‐19 pandemic outbreak and the seldom evidence to the physical, chemical, and environmental variables in these milks in Kuwait.

## 2 MATERIALS AND METHODS

### 2.1 Collection of samples

Milk samples of camel, cow, and goat (each, 30 numbers) were collected by direct milking in sterile glass bottles to avoid potential contamination due to metallic containers from a major farm outlet in Kuwait. The samples were transferred from the farm in an icebox to the laboratory within six h and stored at 4°C. Samples (250 g) were weighed and frozen in the freeze dryer (Labconco FreeZone 18) at −50°C and vacuum applied at 133 × 10^−3^ mbar for 48 h. After the freeze‐drying cycle, the containers were sealed and stored at 5°C and analyzed following the method described by Ibrahim and Khalifa (2015). Freeze‐drying augmented in longer shelf‐life preservation of the solid over the liquid state of milk (Ibrahim & Khalifa, 2015).

### 2.2 Physical and chemical analyses

The collected fresh milk samples were analyzed for pH and conductivity using Fisher Scientific Accumet Research AR50 meter and titratable (total) acidity by following the standard method (AOAC, 2000). Acidity is measured in percentage of lactic acid (Equation (1)). Because 1 ml of 0.1 N lactic acid contains 0.009 g of lactic acid, multiplying the volume of 0.1 N NaOH required to neutralize the lactic acid in the sample by 0.009 will yield the amount of lactic acid (grams) in the sample. This is divided by the weight of the milk sample and multiplied by 100 to obtain the percentage of lactic acid (AOAC, 2000). The specific gravity of the samples followed the gravimetric method by weighing the known measure of milk. Moisture content (Equation (2)) was determined from the loss of mass freeze‐drying in the Labconco‐FreeZone18 Freeze Dryer (Ibrahim & Khalifa, 2015, Valentina et al., 2016). The milk samples were subjected to a prefreezing temperature between −15 and −23°C at 1.65 and 0.67 mbar vacuum set point, respectively, in the freeze dryer. The loss of weight was calculated to determine the moisture content of the sample following the laboratory manual of Labconco‐Freezone18 and method described earlier (Valentina et al., 2016). The freeze‐dried samples were analyzed for ash content (muffle furnace—Carbolite AAF 1100) by the Association of Official Agricultural Chemists (AOAC) (2000) method. Calorific content (1 g sample) was determined by a bomb calorimeter (Ujor et al., 2014).

#### 2.2.1 Determination of trace metals

Open digestion method was applied for the preparation of the samples (1 g) with the acid mixture (10 ml) of HNO_3_:H_2_SO_4_ (3:1) following the method described by Oreste et al. (2016) and the United States Environmental Protection Agency (USEPA) (2014). The digested sample was cooled and allowed to settle before analysis. The essential metals (K, Ca, Na, Mg) and lesser essential trace metals (Ag, Al, As, Ba, Cd, Cu, Fe, Mn, Mo, Se, V, Zn) were determined using inductively coupled plasma‐atomic emission spectrometry (ICP‐AES – PerkinElmer Optima 7300 DV) and inductively coupled plasma‐mass spectroscopy (ICP‐MS – PerkinElmer's NexION 2000), respectively. Along with the investigated samples, quality control (QC) samples were also checked. The analytical detection limits of instrumentation were 0.01 ppm for ICP‐AES and 0.001 ppm for ICP‐MS. The sulfur content was analyzed by Elemental Analyzer (ElementarVario Micro Cube), which was compliant to the Dumas dry combustion technique (ASTM, 2017). Mercury concentrations in the sample were analyzed by Mercury Analyzer (Hydra IIAA) (USEPA 2014).

#### 2.2.2 Determination of nitrogen and protein

The nitrogen contents of crude protein (CP), true protein (TP: nitrogen‐associated protein minus the nonprotein sources), NP nitrogen (NPN) contents were determined following the standard Kjeldahl methods (DeVries et al., 2017; ISO, 2016; ISO, 2016; Lynch & Barbano, 1999). Minor changes to this method to meet accuracy followed the lyophilized milk samples (1 g) digested in the Kjeldahl digester (Gerhardt–Kjeldatherm) in the presence of Kjeldahl digestion tablet (catalyst) with the oxidizing agent namely, conc. H_2_SO_4_ (12 ml) and H_2_O_2_ (6 ml) for 1 h at 200°C and another 1 h at 380°C. The digested sample was diluted with distilled water (75 ml). Ammonia was steam distilled from the digested sample. To this, 50% NaOH (50 ml) solution was added using the Kjeldahl distillation unit (Gerhardt–Vapodest 300). The distillate was collected in a conical flask containing 4% boric acid (50 ml) with two drops of methyl red indicator. The ammonia trapped in boric acid was determined by titration with 0.1N HCl with endpoint color change from red to yellow. Trichloroacetic acid (TCA) (15%–40 ml) was added to the 10 ml of the reconstituted samples (5–10 g) in warm water at 40°C. The solution was settled (5 min) and the formed precipitate (true protein) was filtered through Whatman No. 1 filter paper. The total nitrogen content of a weighed aliquot (up to 20 ml) of filtrate (nitrogen–nonprotein (N–NP)) was determined by the Kjeldahl assay (AOAC, 2012). Quality assurance followed the above determination on all reagents (blanks). The total amount of nitrogen (%N) in the respective milks was calculated using the factors of 6.25, and 14.0067 for CP, TP, and NP (Equations ((3), (4) )), respectively (AOAC, 2012). The nonprotein nitrogen (%NPN) composed of urea, amino acids, uric acid, creatine, creatinine, and ammonia in the milk samples was calculated following the standard method (AOAC, 2012) as indicated (Equation (5)).

#### 2.2.3 Calculations

(1) Acidity%=%Lacticacid

$$\%\;\text{Lactic}\;\text{acid}=\frac{\text{No}.\;\text{of}\;\text{milliliters}\;\text{of}\;0.1\;N\;\text{NaOH}\;\text{solutions}\;\text{required}\;\text{for}\;\text{neutralization}\times 0.009}{\text{Weight}\;\text{of}\;\text{the}\;\text{sample}}\times 100$$

Weight of the sample = Volume of milk × specific gravity. 0.1 N lactic acid contains 0.009 g of lactic acid.

(2) $$\text{Moisture}\;\text{content}\;\left(\text{MC}\right)=\left((W‐d)/W\right)\times 100\%$$

*W*, Wet weight; *d*, Weight after drying.

Nitrogen (with protein in milk) follows the three‐step procedure:

- moles of acid = molarity of acid × volume used in flask (moles A = M × V).
- moles of base = molarity of base × volume added from burette (moles B = M × V).
- subtracting the “moles of base” added from the “moles of acid” gives “moles of ammonia” from the protein, the number of “moles of ammonia” is the same as the “moles of nitrogen,” Thus, grams nitrogen = moles nitrogen × atomic mass (g N = moles N × 14.0067).

(3) %Nitrogen=(gN/gS)×100

g: grams, N: nitrogen, S: sample.

(4) Crudeprotein(CP)=%N×6.25

Conversion factor 6.25 corresponds to the mean nitrogen content of 16% in the pure protein.

(5) Fornonproteinnitrogen(NPN)

$${N‐\mathrm{NP}‐\mathrm{Sample}}_{\%}=\left({N‐\mathrm{NP}‐\mathrm{Filtrate}}_{\%}\right)\times P.F\;\times D.F$$

$$P.F\;\left(\mathrm{Protein}\;\mathrm{factor}\right)=\;\frac{{\mathrm{Mass}\;\mathrm{of}\;\mathrm{test}\;\mathrm{portion}}_{\left(g\right)}+{\mathrm{Mass}\;\mathrm{of}\;\mathrm{TCA}\;\mathrm{solution}\;}_{\left(g\right)}}{{\mathrm{Mass}\;\mathrm{of}\;\mathrm{test}\;\mathrm{portion}}_{\left(g\right)}}$$

$$D.F\;\left(\mathrm{Diluent}\;\mathrm{factor}\right)=\;\frac{{\mathrm{Mass}\;\mathrm{of}\;\mathrm{Sample}}_{\left(g\right)}+{\mathrm{Mass}\;\mathrm{of}\;\mathrm{DI}\;\mathrm{water}\;}_{\left(g\right)}}{{\mathrm{Mass}\;\mathrm{of}\;\mathrm{Sample}}_{\left(g\right)}}$$

#### 2.2.4 Statistical analysis

All the data were analyzed in triplicate. The data were treated using descriptive statistics (Dhanalakshmi & Gawdaman, 2013; Ibrahim & Khalifa, 2015). The results of the physical parameters were incorporated in Section 3.1, while they were transformed to logarithmic values to display the wide‐ranged differential units and reduce the dispersed numerical data values to visualize or respond to skewness toward large values and show the figure in compactness. Additionally, ANOVA was used to test the significant differences between the variables and the samples (Table 1).

**TABLE 1:** Analysis of variance (ANOVA) tests on the physical–chemical parameters of three milk samples

Source of variations	SS	*df*	*F*	*p*‐Value	*F*‐critic
A. Physical parameters					
Physical parameters	14.244	7	798.169	.0001	2.76*
Sampled species	0.001	2	0.152	.86	3.73**
Error	0.036	14			
Total	14.280	23			
B. Biochemical parameters					
Biochemicals	4.57	4	116.825	.000	3.838*
Sampled species	0.02	2	0.824	.473	4.459**
Error	0.08	8			
Total	4.66	14			
C. Major metals					
Heavy metals	1,432,897.04	3	300.15	<.01	4.75*
Species	15,243.07	2	4.78	.057	5.14**
Error	9547.730	6			
Total	1,457,687.85	11			
D. Minor metals					
Trace metals	24.90	10	19.76	<.01	2.34*
Species	0.053	2	0.21	.81	3.49**
Error	2.48	20			
Total	28.25	32			

Abbreviations: *df*, degree of freedom; *F*, *F* value; *F*‐critic: table value, Species: three milk samples, *significant, **insignificant; SS, sum of squares.

## 3 RESULTS AND DISCUSSION

### 3.1 Physical characteristics

**FIGURE 1:**
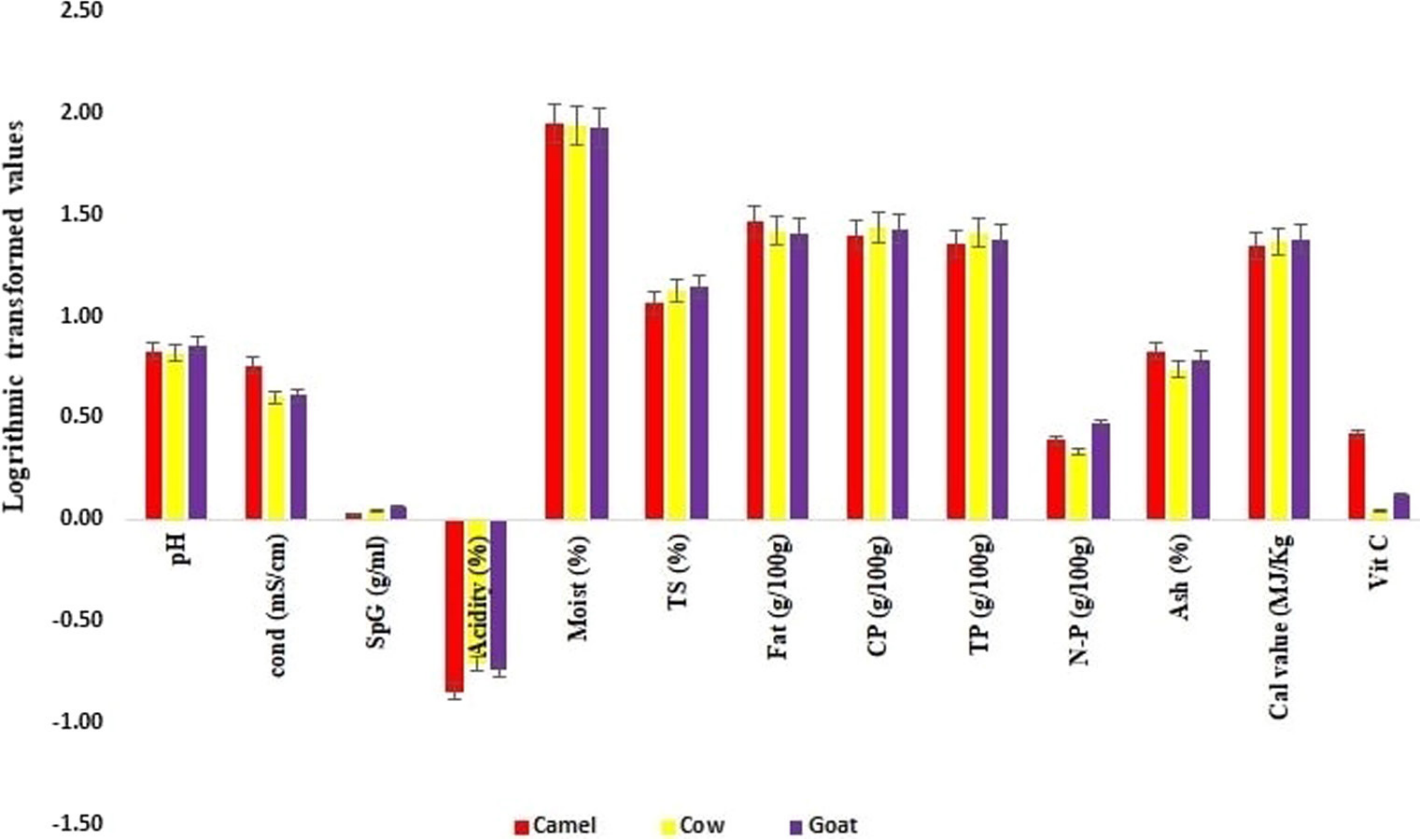
Log‐transformed data of the physical and biochemical parameters in milk samples. Cond, conductivity; SpG, specific gravity; Acid., acidity; Moist, moisture; T.S, total solids; CP, crude protein; TP, true protein; NP, nonprotein; Cal. Val, calorific value

According to the observations, the parameters assessed for the camel, cow, and goat samples showed significant variations (Figure 1). A high pH sequence was found in goats (7.22), camels (6.71), and cows (6.63). In addition to previously reported facts such as higher protein, fat content, and a different arrangement of phosphates compared to other milks, the unusually high alkalinity in fresh goat's milk in this study compared to the pH of goat milk elsewhere can be attributed to the high temperature, high minerals’ content in the halophilic desert plants it eats, and hard water it drinks (Alcantara et al., ; El‐din, 2012; Hossain & Dev, 2013; Ibrahim & Khalifa, 2015; Legesse et al., 2017).

The conductivity of the three milk samples ranged from 4 to 5.8 mS/cm, with the cow milk (4.00 mS/cm) having the lowest conductivity, followed by goat milk (4.10 mS/cm and camel milk (5.80 mS/cm) having the highest conductivity. The varied types and quantities of electrolytes present in these milk samples may account for the differential conductivity (Henningsson et al., 2005). The variant‐specific gravity in the milk samples (Figure 1) reflected the presence of water content derived from feed, body constituents, the type of breed, age, and gender of the animals (Ping et al., 2014; Sabahelkhier et al., 2012).

Milk samples had moisture levels that ranged from 85.78% to 88.28%. The low to high moisture content was reported in the sequence of goat > cow > camel milk among the tested milk samples (Figure 1). Their percentages ranged from 80% to 90%, which was like the previous findings (Mohsin et al., 2019). The other components in milk are suspended in colloidal suspension in water, which serves as a medium for the solution.

Total solids in milk samples ranged from 11.72 to 14.22%, which included fat and nonfat components (Figure 1). In camel, cow, and goat milk, there was a progression of low to high concentrations. The low total solids’ contents are attributable to an increase in water content in the milk caused by thirsty camels consuming too much water. This was consistent with previous research (Sabahelkhier et al., 2012). Acidity ranged from 0.14 to 0.20% (Figure 1). This level corresponded to the previous findings (Yang et al., 2013). Although the statistical test of ANOVA revealed significant differences between the different physical parameters and the three milk samples (Table 1‐A), the variations within the milk of each sample were found insignificant (Table 1‐A).

### 3.2 Biochemical constituents

The amount of CP, TP, NP, fat, vitamin C, calorific value, ash, and trace metals in the milk samples was also measured using various chemical analyses, as shown in Figure 1. Chemical characteristics of samples varied widely, and they excelled in one or more aspects. The log‐transformed value of the CP (25.15 g/100 g to 27.65 g/100 g = 1.40 to 1.44), TP (22.68 g/100 g to 25.5277 g/100 g = 1.355 to 1.40), and NP (2.13 g/100 g to 2.92 g/100 g = 0.33 to 0.47) proved that milk contains more protein than other elements. The fat content (log‐transformed value) was found to be low in the goat milk (25.77 g/100 g = 1.411), compared to cow's milk (26.57 g/100 g = 1.42) and camel milk (29.76 g/100 g = 1.47). This is due to a dilution effect caused by an increased goat milk volume until lactation peak, as well as a decrease in lipid mobilization, which reduces plasma nonester fatty acid availability (ISO, 2016; Lopez et al., 2019). Camel's milk showed high vitamin C (9.89 g/100 g), followed by goat's milk (7.98 g/100 g) and cow's milk (2.09 g/100 g), demonstrating their impact of nutrition. The total energy calorific values in camels, cows, and goats were 5.38 × 10^−6^ kcal/kg, 5.61 × 10^−6^, and 5.76 × 10^−6^, respectively. Furthermore, despite residents of a particular country's preference for distinct tastes, this study indicated not only the possibility of replacement of camel's milk over other milk, but also attributed in line with earlier studies (El‐Agamy et al., 1992; Shamsia, 2009; Tahereh & Hussain, 2021) that characterized high calorific value, nutritional composition, richness in lactoferrin (natural immune booster), low sugar, significant antioxidant and antimicrobial properties that is the need‐of‐the‐hour to augment immunity during the present Covid‐19 pandemic outbreak. Statistical test of ANOVA revealed significant differences between the different biochemical parameters, and the three milk samples but, insignificant within each sample species (Table 1‐B).

### 3.3 Inorganic constituents

**FIGURE 2:**
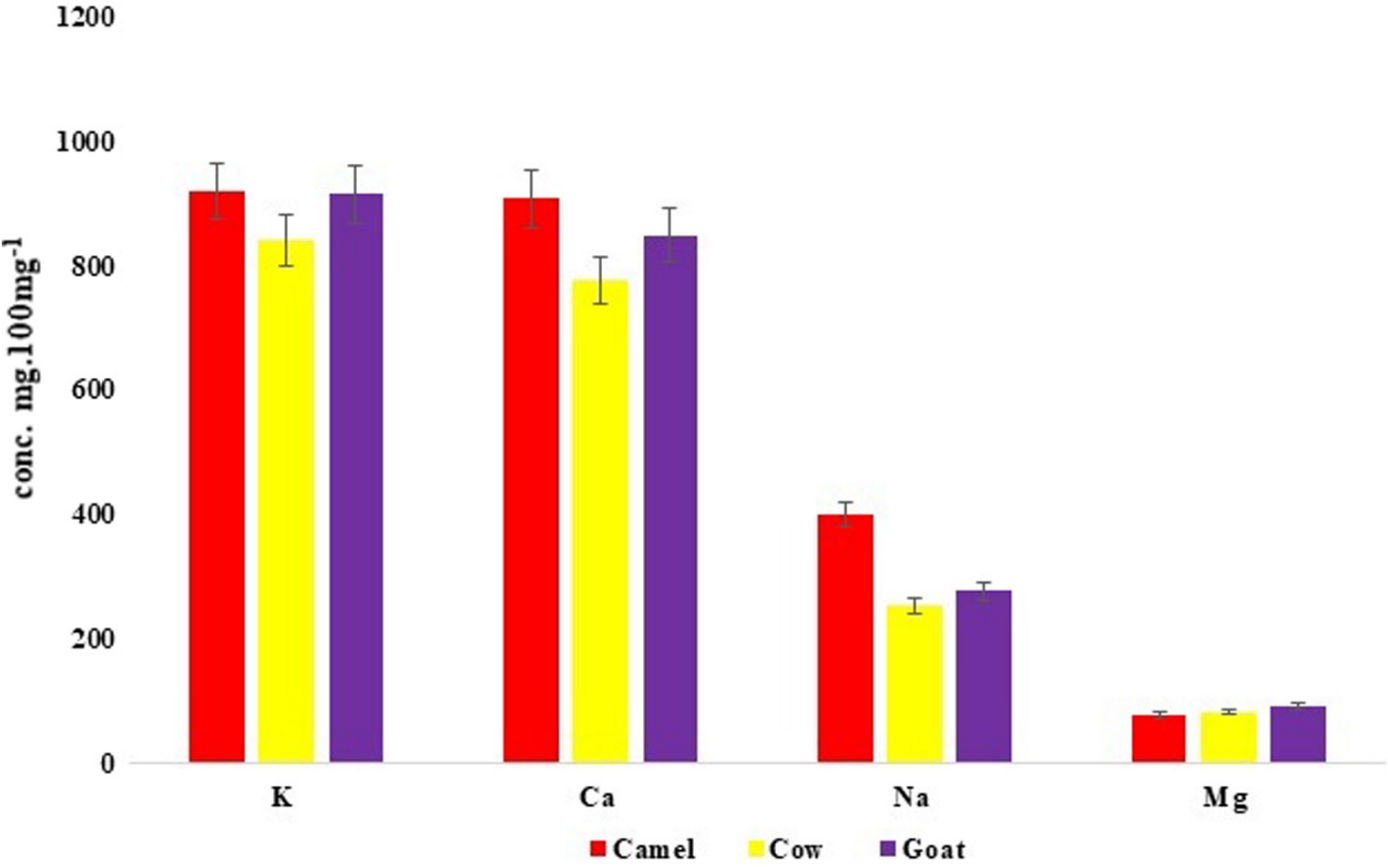
Major heavy metals’ concentrations in the three milk samples

**FIGURE 3:**
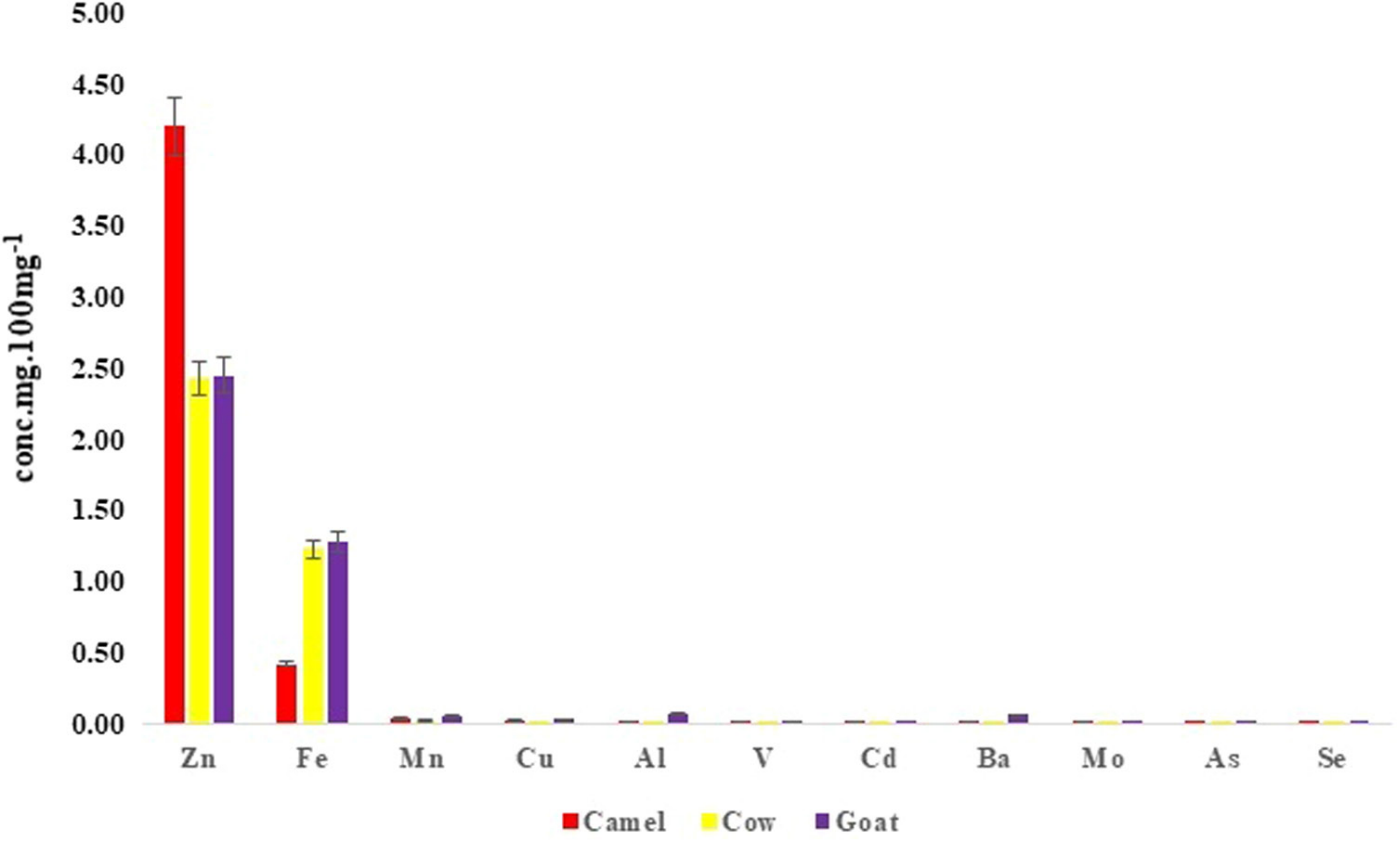
Minor trace metals’ concentrations in the three milk samples

Metals of importance in the milk samples were analyzed in the ICP‐MS (Figures 2, 3). The concentrations of Na, K, Ca, and Zn were high in camel, compared to their concentrations in goat and cow (Figure 2). This indicated the medicinal properties and transfer of minerals from herbs in camel milk and which were found to be in line with the earlier findings (Chirinos‐Peinado & Castro‐Bedriñana, 2020; Kaskous, 2016; Rasheed, 2017). However, Mg was found to be high in goat's milk compared to cow and camel milk. This validated the superior nutritional characteristics in goat's milk, as described by Zenebe et al. (2014), Dhanalakshmi and Gawdaman (2013). The increase in lead (Pb) and cadmium (Cd) concentrations has drawn the attention of researchers, since Pb is known to disturb the effects on brain development (Mason et al., 2014; Pilarczyk et al., 2013; Sarsembayeva et al., 2020). Furthermore, the concentrations of Pb and Cd in milk showed serious dietary constituent concern to infants and children. This agreed with the earlier research of Ali and Khan (2019) and Miclean et al. (2019). However, the Cd concentration in the cow's and goat's milk was below detectable limits, except in the camel milk (0.009 ± 0.0009 mg/100g). Few trace metals that were harmful to human health (Figure 3) were found below the detectable and global permissible limits (Ayangbenro & Babalola, 2017; Chirinos‐Peinado & Castro‐Bedriñana, 2020; ISO, 2016). ANOVA test revealed significant differences between the heavy metals and trace metals, irrespective of the three milk samples’ analysis (Table 1‐C and D). The insignificant differences within the three milk samples indicated that there was no interspecies’ inorganic constituent relationship thus, attributed to the variations met within each milk sample (Table 1‐C and D). This was in line with the earlier studies of Chen et al. (2020), DeVries et al. (2017), Singh et al. (2017), Turkmen (2017), Zibaee et al. (2015), Dhanalakshmi and Gawdaman (2013), describing the varying factors that characterized prefreeze and postfreeze drying, and pasteurization of milk governing the chemical dynamics in their body constituents. Nevertheless, statistical tests showed significant correlation coefficient between the physical, biochemical, and inorganic constituents (Table 2) of the three milks indicating the nature of these animals sharing similar kinds of fodder in nature.

**TABLE 2:** Correlation coefficient between physical, biochemical, and inorganic constituents in the three milk samples

Milk	Description	Camel	Cow	Goat
Camel	Pearson correlation	1	**0.995** ^a^	**0.996** ^a^
	Sig. (2‐tailed)		0.000	0.000
	*N*	27	27	27
Cow	Pearson correlation	**0.995** ^a^	**1**	**1.000** ^a^
	Sig. (2‐tailed)	0.000		0.000
	*N*	27	27	27
Goat	Pearson correlation	**0.996** ^a^	**1.000** ^a^	1
	Sig. (2‐tailed)	0.000	0.000	
	*N*	27	27	27

^a^ Correlation is significant at the 0.01 level (2‐tailed).

Bold value represents significant difference at 0.01 *p*‐value

## 4 CONCLUSION

The exposition on the analysis of less commonly ingested camel milk found distinct characteristics in terms of immune booster for the health of Kuwait's residents. In view of the recent Covid‐19 pandemic outbreak, the unparalleled physical, biochemical properties and chemotherapeutic value of camel's milk inherited from antioxidant nutritionally rich desert plants were found to generate a strong immunity in normal healthy residents as well, and to combat the ailment in patients with respiratory ailments. Furthermore, the comparative analyses validated the supreme qualities of camel milk such as the action of high lactoferrin, calorific value, and antimicrobial properties over the cow's and goat's milk. Additionally, this study recommends regular monitoring and analysis of these milks for safe consumption, since the camel milk is consumed raw, unlike the consumption of other milk by the residents.

## CONFLICT OF INTEREST

The author declares that she has no conflict of interest.

## PATIENT CONSENT

Not applicable.

## ETHICS APPROVAL

All study methods were carried out in accordance with relevant guidelines and regulations. As this study has not involved in any human and animal subjects, the author declares no applicability of ethical standards. Local guidelines followed as per the Kuwait University direction.

## PERMISSION TO REPRODUCE

Following as per the guidelines of publisher acceptance.

## Data Availability

The data that support the findings of this study are available on request from the corresponding author. The data are not publicly available due to privacy, ethical, and commercial restrictions made in agreement with the farm owners who generously supplied milk for this study.
